# Rashba spin-orbit coupling in graphene monolayer coated by periodic magnetic stripes

**DOI:** 10.1038/s41598-017-06821-9

**Published:** 2017-07-26

**Authors:** Xiaojing Li, Zhenhua Wu, Jiangtao Liu

**Affiliations:** 1 College of Physics and Energy, Fujian Normal University, Fuzhou, 350007 China; 2 Key Laboratory of Microelectronic Devices and Integrated Technology, Institute of Microelectronics, Chinese Academy of Sciences, Beijing, 100029 P.R. China; 3 Nanoscale Science and Technology Laboratory, Nanchang University, Nanchang, 330031 China

## Abstract

We investigate theoretically the effects of a modulated periodic perpendicular magnetic fields and the Rashba spin-orbit coupling (RSOC) on the electronic states and optical absorption spectrum in a graphene monolayer. The magnetic fields and supperlattice geometry give rise to distinct Dirac cone shift and open a finite bandgap at the Dirac point. In contrast to the energy spectrum without the RSOC interaction, we find that the RSOC term will develop a spin-splitting energy-momentum dispersion relation in this graphene magnetic supperlattice. Anisotropic and spin-split group velocities, effective masses and the momentum-dependent carrier distributions along the magnetic strips are demonstrated. And the manipulations of these exotic properties by tuning the magnetic fields and the RSOC are addressed systematically. Accordingly, we find bright-to-dark transitions in the electron-hole pairs transition rate spectrum and absorption peak splitting in the optical absorption spectrum tuned by the RSOC interaction. This feature offers us a practical way to detect these band engineering effects especially the exotic spin splitting states by using the conductance and optical technique.

## Introduction

Graphene monolayer and few layers have been created by mechanical exfoliation of small mesas of graphite, CVD, graphite oxidation reduction and other chemical synthesis methods^1^, especially the monolayer graphene has the most impressive two-dimensional electron gas properties, including long phase coherence lengths, strong electric field responses, Dirac fermions behaviors with no energy dissipation, which promise a great potential for the applications in the nano-electronics. In addition, within a very large wavelength range, graphene possesses large and almost-uniform absorption coefficients but small optical absorptance. The small optical absorptance makes graphene an attractive alternative for optoelectronic devices such as transparent electrodes and optical display materials^2–4^. People have studied the dynamics of the electrons in a nonuniform magnetic field applied perpendicular to a bilayer graphene sheet and showed that snake states in graphene exhibit peculiar properties related to the underlying dynamics of the Dirac fermions^5^. However, making high quality graphene based devices involves etching it into nanoscale structures, creating commensurate potentials on the lattice scale with acceptable disorders, *et al.*, which are very experimentally challenging. Therefore the prospect of employing graphene as a building block in optoelectronic devices has stimulated an intense research activity addressing the problem of how to manipulate its peculiar electronic states in a practical way that is robust to the disorders. Proposal of synthetic superlattices by Esaki and Tsu by combining alternate nano-layers of materials^6^ offers us new way for band engineering and charge flow controlling. Recently a great deal of attention has been devoted to superlattice structures, where external spatially periodic electric^7–12^ and/or magnetic fields are applied to a graphene monolayer^13–16^. In these previous studies, people have found anisotropic energy dispersion and gap modulation by the external fields. However the optical response and transport property of such magnetic superlattice system have not been investigated thoroughly until now, especially for the experimental measurable quantities, like conductance, optical absorption spectrum. Additionally the hybrid impact of the periodic magnetic stripes in coexistence with the Rashba spin-orbit coupling (RSOC) has not yet been reported or fully understood in these researches.

The Rashba spin-orbit coupling(RSOC) is a relativistic effect. The RSOC in graphene arising from crystal with structural inversion asymmetry by impurities or external electric fields has been theoretically predicted^17, 18^ and experimentally observed^19^. Compared to the relatively small intrinsic SOC in graphene, the RSOC can be greatly enhanced as experimentally demonstrated in recent work with the order of 10 meV^20^. In crystals without an inversion center, electric energy bands are split by RSOC under the modulation of doped impurities or an external electric field. The exploration of the RSOC, including both physics and applications, is now a rapidly growing research field of spin-orbitronics^21^, a new direction of spintronics focusing on the manipulation of non-equilibrium materials properties using the RSOC.

In this work, we propose a magneto-optical instrument utilizing a graphene magnetic superlattice which can be realized by coating a periodic array of ferromagnetic stripes above it. we demonstrate a clear conception about the graphene monolayer under periodic magnetic stripes, which leads to a magnetic superlattice in graphene without the need for cutting or etching. We investigate the effect of a periodic magnetic field and the RSOC interaction on the energy dispersions and corresponding transport properties, like group velocity and effective mass. The longitudinal propagation of charge carriers through such a graphene magnetic superlattice can be tuned significantly by changing the incident Fermi energies, magnetic fields and the superlattice structures. The transverse propagation is highly anisotropic. The electrons of opposite wave vectors (±*k*_*y*_) tend to spatially separated distributions in a superlattice cell. A bright-to-dark transition in optical transition rate of e-h pairs can be tuned by the RSOC interactions. We can also find the highly anisotropic behavior in transition rate spectrum and spin splitting states in optical absorption.

## Methods

**Figure 1:**
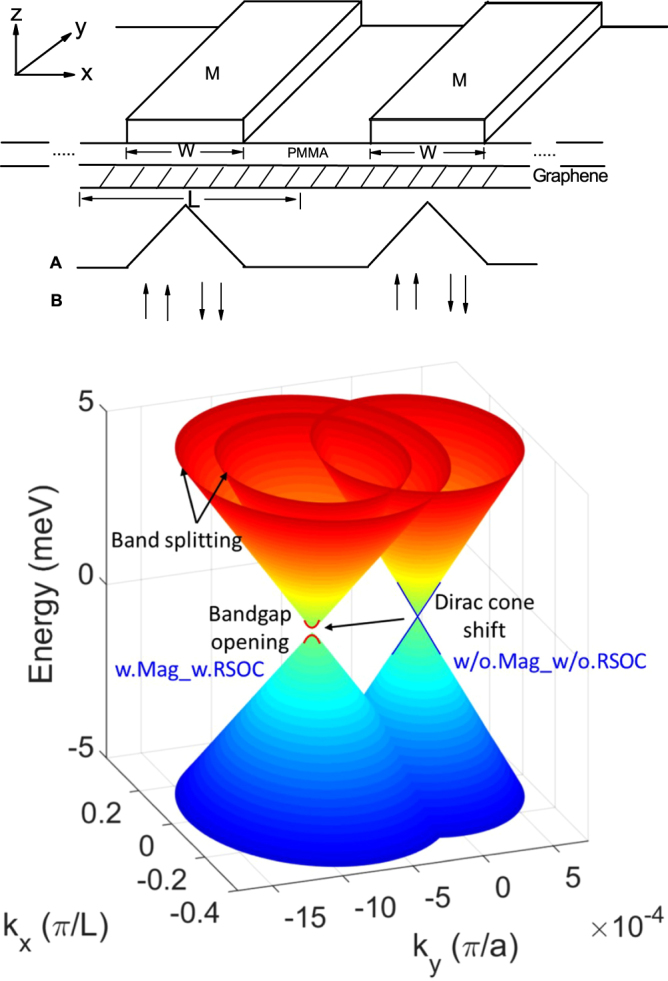
(Top) Schematic diagram of a graphene monolayer coated by periodic magnetic stripes and the vector potential profile in the proposed structure. (Bottom) The Dirac cones of a bare graphene layer and a graphene magnetic superlattice in the precense of the RSOC, with *L* = 100 *nm*, *W* = 50 *nm*, *B* = 1*T*, *R*_0_ = 10^2^ *m*/*s*.

We consider a graphene monolayer coated by periodic magnetic stripes along *y* direction as shown in Fig. 1 (Top). The magnetic field is applied perpendicular to the graphene layer. The low-energy quasiparticles in graphene with the RSOC interaction can be well described by the Dirac-like Hamiltonian^22^,

1 $$H={H}_{0}+{H}_{Z}+{H}_{R}={v}_{f}\sigma \cdot ({\bf{p}}+{\bf{eA}})+g{\mu }_{B}\sigma \cdot {\bf{B}}+{H}_{R},$$

where *v*_*f*_ ≈ 10^6^ *ms* ^−1^ is the Fermi velocity. *σ* = (*σ*_*x*_, *σ*_*y*_) are Pauli matrices, **p** = −**i** *ħ*∇ is the momentum operator and vector potential **A** is related to the magnetic field **B** applied perpendicular to the film by ∇ × **A** = **B**. The magnetic strips induce periodic magnetic fields of pointing up and down together. Accordingly the vector potential **A** in each cell are given by:

2 $${\bf{B}}=\{\begin{array}{ll}(0,0,B); & x\in [-\frac{W}{2},0)\\ (0,0,-B); & x\in [0,\frac{W}{2}]\\ (0,0,0); & x\notin [-\frac{W}{2},\frac{W}{2}]\end{array}$$

3 $${\bf{A}}=\{\begin{array}{ll}(0,Bx+\frac{WB}{2},0); & x\in [-\frac{W}{2},0)\\ (0,-Bx+\frac{WB}{2},0); & x\in [0,\frac{W}{2}]\\ \mathrm{(0},0,0); & x\notin [-\frac{W}{2},\frac{W}{2}]\end{array}$$

in which *W* is the width of magnetic stripes. This description is accurate theoretically^7^ and has also been proved experimentally. The second term *H*_*Z*_ = *gμ*_*B*_σ · **B** is induced by Zeeman splitting. The impact of Zeeman term in our calculation is negligibly small with splitting energy of *E*_*Z*_ = 0.12 *meV* at *B* = 1*T*. The structural inversion asymmetry (SIA) terms couples the two blocks of *H*_0_ such that the axial spin symmetry is broken. Here, we include only the most important RSOC term^23^ which is linear in momentum giving rise to the following Hamiltonian matrix for the *K* valley in the basis |*A* ↑ 〉, |*B* ↑ 〉, |*A* ↓ 〉, |*B* ↓ 〉. A and B correspond to the sublattice of graphene and the arrows denote the electron spin,

4 $${H}_{R}=[\begin{array}{cccc}0 & 0 & -i{R}_{0}{p}_{-} & 0\\ 0 & 0 & 0 & -i{R}_{0}{p}_{-}\\ i{R}_{0}{p}_{+} & 0 & 0 & 0\\ 0 & i{R}_{0}{p}_{+} & 0 & 0\end{array}].$$

Note that the electronic properties of graphene are invariant by interchanging the *K* and *K*′ states. We can use this reduced 4 × 4 Hamiltonian since the matrix for both *K* and *K*′ valleys is block-diagonal with degenerated states. The Rashba term in *H*_*R*_ only couples the electrons from the same valley. The value of the RSOC parameter *R*_0_ depends on the external electric field perpendicular to the graphene plane. We take *R*_0_ = 10^2^ *m*/*s* for the calculations^21^. To solve the single particle problem we assume that the graphene structure is embedded in a large hard wall rectangular box with the sizes *L*_*i*_ (*i* = *x*, *y*). The electron wave functions are expanded in a plane wave basis confined by the large hard wall box. The wave function Ψ for electron can be expanded as

5 $${\rm{\Psi }}({k}_{x},{k}_{y})=\sum _{n}{{\bf{C}}}_{n}{\varphi }_{n}({k}_{x},{k}_{y})=\sum _{n}{{\bf{C}}}_{n}\frac{1}{\sqrt{L}}{e}^{i(\frac{2n\pi x}{L}+{k}_{x}x)}{e}^{i{k}_{y}y},$$

where *k*_*x*_ (*k*_*y*_) is the wave vector in the x (y) direction, and the expansion coefficient **C**_*n*_ a four-component column vector. The wave function can be calculated numerically in the basis set with the periodic boundary conditions in the *x* (*y*) direction.

Based on this Hamiltonian we denote the velocity operator of the carrier as $\overrightarrow{v}(k)=\frac{i}{\hslash }[H,\overrightarrow{r}]={v}_{f}({\sigma }_{x},{\sigma }_{y})$. In our calculation the Fermi velocity *v*_*f*_ = 0.86 × 10^6^ *ms* ^−1^ is used^24^. The effective mass is defined as the ${m}_{ij}={\hslash }^{2}{(\frac{{\partial }^{2}E}{\partial {k}_{i}\partial {k}_{j}})}^{-1}$. We also calculate the conductance of a magnetic barrier and a superlattice by employing the Landauer-Büttiker formalism^25, 26^,

6 $$G({E}_{F})={G}_{0}{\int }_{-\infty }^{\infty }{\int }_{-{k}_{F}}^{{k}_{F}}T({E}_{F},{k}_{y}){F}_{T}(E-{E}_{F})dEd{k}_{y},$$

where ${G}_{0}\equiv \frac{{e}^{2}{L}_{y}}{\pi h}$ is taken as the conductance unit, *L*_*y*_ is the sample size along the *y* direction which is much larger than *L* and *a*, *F*_*T*_(*E* − *E*_*F*_) = −*df*(*E*)/*dE* is the thermal broadening function, and *f*(*E*) is the Fermi-Dirac distribution function.

The interaction Hamiltonian between the Dirac fermion and the photon within the electrical dipole approximation is ${H}_{int}=H(\overrightarrow{p}+e\overrightarrow{A})-H(\overrightarrow{p})$ ^27^, where the vector potential $\overrightarrow{A}=({\overrightarrow{A}}_{x}\pm i{\overrightarrow{A}}_{y}){e}^{-i\omega t}$ corresponds to the *σ*± circularly polarized light. |*i*〉 denotes the initial states in the lower cones that are hole or valence like, |*f*〉 denotes the final states in the upper cone states that are electron of conduction like. Now the electron-light interaction induces transition from |*i*〉 to |*f*〉, |*i*〉 and |*f*〉 are written as Ψ_*e*,*h*_(*k*_*x*_, *k*_*y*_) in Eq. 5. The resulting optical transition rate of e-h pair between valence and conduction band is $|\langle f|{H}_{int}|i\rangle |$. The transition rate is given by ref. 28,

7 $${w}_{if}=2\pi \delta ({E}_{f}-{E}_{i}-\hslash \omega ){|\langle f|{H}_{int}|i\rangle |}^{2}.$$

in which $\langle f|{H}_{int}|i\rangle ={\sum }_{n,m}{{\bf{C}}}_{f,n}^{+}{\varphi }_{f,n}^{\ast }{H}_{int}{{\bf{C}}}_{i,m}{\varphi }_{i,m}$. Finally we can obtain the optical absorption rate by the integral of transition rates in *k* space and

8 $$\alpha (\hslash \omega )={\iint }_{{k}_{x},{k}_{y}}\sum _{i,f}{w}_{if}d{k}_{x}d{k}_{y}\mathrm{.}$$

## Discussion

### Energy Spectrum

**Figure 2:**
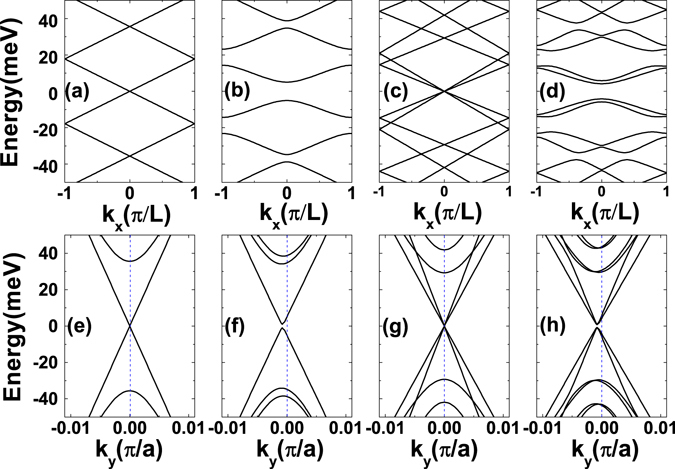
(**a–d**) The energy dispersions as a function of the wave vector *k*_*x*_(in units of *π*/*L*) when *k*_*y*_ = 0. (**a**) *B* = 0, *R*_0_ = 0; (**b**) *B* = 1*T*, *R*_0_ = 0; (**c**) *B* = 0, *R*_0_ = 10^2^ *m*/*s*; (**d**) *B* = 1*T*, *R*_0_ = 10^2^ *m*/*s*. The corresponding energy dispersions as a function of the wave vector *k*_*y*_ are plotted in (**e–h**) when *k*_*x*_ = 0.

In our calculation the *x* direction has a varying character where the graphene sheet is alternately uncovered or covered by a ferromagnetic strip of *W* = 50 *nm*, creating one unit cell with period of *L* = 100 *nm* as sketched in Fig. 1 (Top). The *y* direction is uniform with period of *a* = 0.246 *nm*. The magnetic stripes generate inhomogeneous magnetic fields cooperating with the RSOC, that change the band structure of graphene significantly as shown in Fig. 1 (Bottom). The RSOC breaks the axial spin symmetry and thus the single Dirac cone could split into double Dirac cones. The external magnetic fields break the time-inversion symmetry giving rise to a shift of the Dirac cone in the reciprocal space. A bandgap develops at the Dirac point arising from the hybrid effects of the magnetic fields and the superlattice structures. It enables more practical ways to tune the the electronic and optical properties of graphene. First, we illustrate the energy spectrum *E*(*k*_*x*_) of the graphene magnetic superlattice in Fig. 2(a–d). For a bare graphene layer, the energy dispersion exhibits a linear dependence on wave vector *k*_*x*_ near the Γ point (*k*_*x*_ = *k*_*y*_ = 0). When a finite magnetic field is applied by the periodic magnetic stripes, the energy spectrum becomes parabolic as shown in Fig. 2(b). A finite gap of about several meV can be achieved with a magnetic field of 1 Tesla. In Fig. 2(c) we show the impact of the RSOC induced by a perpendicular electric field without an external magnetic field. The RSOC term couples the spin-up and spin-down electrons in the same valley as denoted in employed model, which can lift the spin degeneracy of the electrons. The spin-up and the spin-down electrons develop split Dirac cones with different group velocities and distributions, and thus spin filtering and detection become feasible. Note that the RSOC cannot open a bandgap near the Γ point because the strength of the RSOC term is proportional to the wave vector **k** and vanishes when **k** = 0. When both a magnetic field and an electric filed are applied on the graphene layer, we can observe both parabolic energy dispersion and spin states splitting as shown in Fig. 2(d). Compared with Fig. 2(b), the spin splitting gives rise to a bandgap shrinkage as shown in Fig. 2(d). The effect of the RSOC is contrary to the magnetic field on bandgap manipulation and in competition with each other.

Similarly we also plot the energy spectrum of the graphene magnetic superlattice as dependence of *k*_*y*_ as shown in Fig. 2(e–h). The energy dispersion relation of the ground state is linear as respect to the wave vector *k*_*y*_ when the wave vector *k*_*x*_ and the magnetic field are both zero, see Fig. 2(e). When a periodic magnetic field is applied by the top magnetic stripes, energy spectrum is no longer symmetrical. The Dirac cone is shifted away from the Γ point as shown in Fig. 2(f). The shift distance is determined by the strength of magnetic fields via the vector potential in the Hamiltonian, see Eq. 1. The strong anisotropy of the energy dispersion indicates that the electrons with negative *k*_*y*_ feel different external fields and group velocities from the ones with positive *k*_*y*_ as we will discuss in more details in subsection conductance. When we take the RSOC term into consideration without a magnetic field, we can find that the effect of the RSOC on the dispersion is quite different from the magnetic field. The degeneracy of excited states is lifted by the RSOC when *k*_*y*_ ≠ 0, which can be verified by the optical measurement as illustrated subsection optical Absoeption Spectrum. The energy spectrum of spin-up carriers splits from the spectrum of spin-down carriers as shown in Fig. 2(g), similarly as Fig. 2(c). When including both the periodic magnetic field and the RSOC term, we can observe not only the splitting of energy spectrum but also the degeneracy lifting of the excited energy bands in Fig. 2(h). Next we will discuss how the band structure modulation impacts on the carrier transport properties and optical properties in the graphene magnetic superlattice.

### Conductance

**Figure 3:**
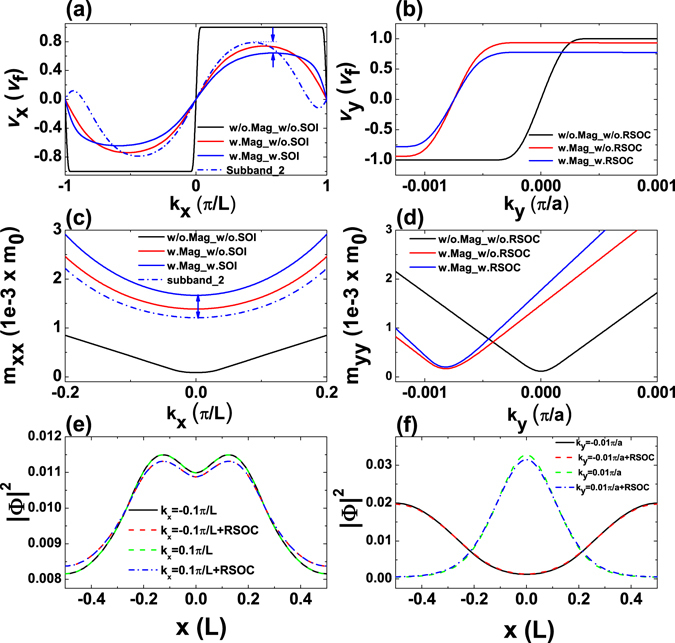
(**a** and **b**) plot the group velocities *v*_*x*_ and *v*_*y*_ of electrons in the ground state or the second subband as functions of momentum *k*_*x*_ and *k*_*y*_ (*B* = 1*T*, *R*_0_ = 10^2^ *m*/*s* in case the magnetic field and/or RSOC term are induced). (**c** and **d**) are the effective masses *m*_*xx*_ and *m*_*yy*_ as functions of *k*_*x*_ and *k*_*y*_. (**e** and **f**) The probability distribution of carriers in the ground electron states with different *k*_*x*_ and *k*_*y*_ in a superlattice cell.

The carrier transport properties in such graphene-based magnetic superlattice can be tuned dramatically according to the band engineering by the periodic magnetic fields as well as the RSOC. In Fig. 3(a), we plot the group velocity of ground-state electrons along the *x* direction (*v*_*x*_) as a function of the wave vector *k*_*x*_ with/without magnetic field and the RSOC term. For an electron in the Dirac cone the group velocity is approaching the Fermi velocity and is almost unchanged with wave vector *k*_*x*_. When a magnetic field is applied, the group velocity exhibits a sinusoidal shape arising from the parabolic energy spectrum as shown in Fig. 2(b). The group velocities of electrons in the graphene magnetic superlattice are always smaller than the Fermi velocity, which is consistent with a heuristic semiclassic picture, i.e., the magnetic fields bend the electrons away from motion direction leading to a reduction in group velocities. When the RSOC is induced, the maximum group velocity is further reduced. Because the RSOC term arising from the external electric field is equivalent to an additional in-plane magnetic field. When the electrons move along the *x* direction, the in-plane magnetic field tries to push the electron out of the plane, resulting in the reduced velocity along the *x* direction. Furthermore, we also calculate the group velocity of the second subband in the presence of both magnetic fields and the RSOC. The maximum velocity of electrons in the second subband is higher than that of ground-state electrons. Note that the first and second subband are spin-split subbands induced by the RSOC. Therefore, spin-polarized electrons in these two subbands are of different velocities. The relative difference, including magnitude and direction, in spin-up and spin-down-electron velocities varies with wave vector *k*_*x*_. This feature makes the graphene magnetic superlattice a proming building block of possible spin/momentum-filter devices. Correspondingly we plot the group velocity of the ground-state electrons along the *y* direction (*v*_*y*_) as a function of the wave vector *k*_*y*_ in Fig. 3(b). In contrast to *v*_*x*_, *v*_*y*_ exhibits a step-like shape and a lateral shift when a magnetic field is applied. The asymmetrical velocity component denotes the relocated Dirac cone and the anisotropic effective fields along *y* directions. Similar behavior of the suppression on *v*_*y*_ by the RSOC is also observed. In Fig. 3(c) and (d) we plot the effective mass along the *x* and the *y* directions (*m*_*xx*_ and *m*_*yy*_). We can find that the effective mass is approaching zero for a bare graphene layer near the band edge. Light effective mass results in high mobility. When a periodic magnetic field is applied, *m*_*xx*_ can be increased effectively. When the RSOC is induced, it further increases the *m*_*xx*_, that is in consistent with the velocity drop. For the second subband, the effective mass *m*_*xx*_ is reduced effectively, indicating a superior electron transport ability with opposite spin polarization. The *m*_*yy*_ shows asymmetrical profile when a magnetic field is applied also due to the introducing of vector potential in the Hamiltonian and the gauge we used. The RSOC term can enlarge effective mass and this effect becomes more significant with increasing the *k*_*y*_ due to the RSOC term ±*R*_0_ *p*_±_ in Eq. 4. Finally we can observe the nonzero anisotropy of effective mass arising from the the collective effects of both magnetic fields and the RSOC term. These features would have distinct impacts on the conductances we will discuss later in this subsection.

In Fig. 3(e) and (f) we plot the probability distributions of electrons in the first conduction band of the graphene magnetic superlattice. The distribution is isotropic for positive and negative *k*_*x*_. The electrons tend to concentrate in the center of a cell, i.e., in the magnetic barrier region. Since the perpendicular magnetic fields develop a circular motion of an electron and prevent it transmitting away. However the distribution is anisotropic for *k*_*y*_, when *k*_*y*_ is negative, the electrons tend to distribute along the edge of a cell where the graphene is not coated with magnetic stripes above. On the other hand, if the *k*_*y*_ is positive, the electrons tend to concentrate in the center of a cell where the graphene is coated with magnetic stripes. It indicates that the carriers moving in opposite (±*y*) directions are spatially separated in a periodic cell of the magnetic superlattice. The magnetic field has apparent effect on the election distributions with different *k*_*y*_. This behavior can be understood by the profile of the effective vector potential in the Hamiltonian, in which the effective vector potential acts as *k*_*y*_-related barrier altered by magnetic field. This behavior can also be interpreted semiclassically as the Lorenz force whose direction depends on the moving direction of electrons. The RSOC term tends to push the electrons out of the plane resulting in the squeezed distributions as shown in Fig. 3(e) and (f). Compared with the probability distributions of electrons with different signs of *k*_*x*_, the distributions of electrons with different sign of *k*_*y*_ are less sensitive to the RSOC term. Note that the strength of the RSOC is adjustable, so it keeps the potential to control the electrostatic properties.

**Figure 4:**
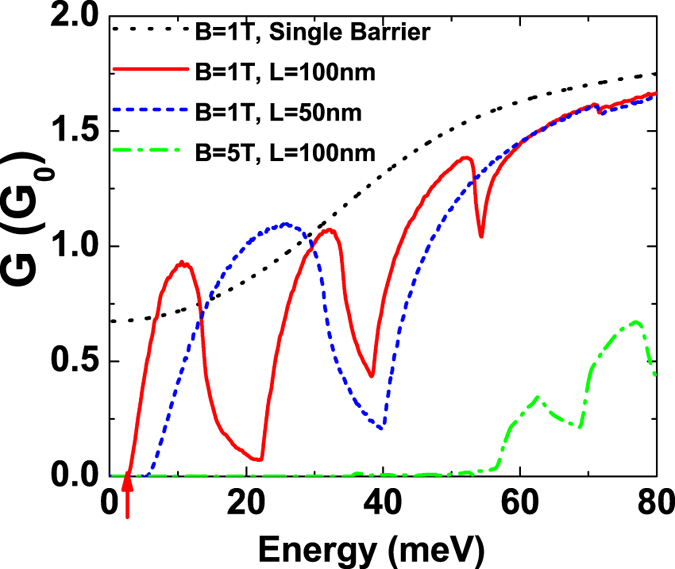
Transport conduction as a function of Fermi energy with different magnetic fields and superlattice period lengths. The red arrow indicates the conductance gap as a manifestation of a finite bandgap.

The conductance is a quantity easier to measure than the group velocity or the effective mass. We have also calculated the conductance *G* accounting for the lowest subband of the graphene based magnetic superlattice structures, using Eq. 6, and compared it with the conductance of only a single magnetic strip. In Fig. 4, we plot *G* as a function of the electron incident Fermi energy. In contrast to the smooth variation of the conductance *G* for a single magnetic strip, *G* for the magnetic superlattice vanishes at the low energy region and turns to oscillate as we increase the energy. The vanished conductance arises from the evanescent modes with an imaginary wave vector in the superlattice. It indicates that a finite band gap is opened, which is consistent with the band structure as we have obtained. The conductance gap can be enlarged by reducing the distance between two adjacent magnetic stripes or by increasing the magnetic fields strength as expected. The oscillations are caused by the Fabry-P *e*′ rot resonant modes formed between two adjacent magnetic stripes due to the multiple reflections at the interfaces^29, 30^. The resonances in *G* are more pronounced for larger distance *L* since more Fabry-P*é*rot modes are formed. We can therefore effectively tune the carrier transport properties in graphene by using the periodic magnetic stripes.

### Optical Absorption Spectrum

**Figure 5:**
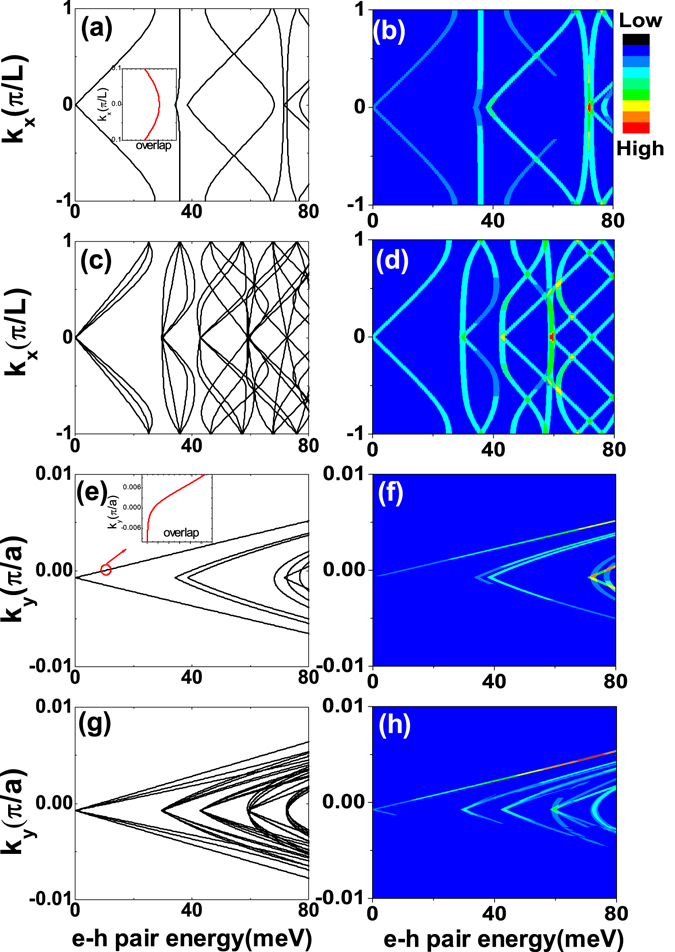
(**a**) The energy dispersions of e-h pairs as a function of wave vector *k*_*x*_ with *B* = 1*T*, *R*_0_ = 0 around the band bottom. (**b**) The optical transition rate of e–h pairs as a function of wave vector *k*_*x*_ *B* = 1*T*, *R*_0_ = 0. (**c** and **d**) are the same as (**a** and **b**) but including the RSOC term *R*_0_ = 10^2^ *m*/*s*. (**e–h**) are the corresponding energy dispersions and the optical transition rate as a function of the wave vector *k*_*y*_.

To monitor the band structure modulation, we investigate the effect of magnetic fields and the RSOC term on the energy and *σ*+ circularly polarized optical transition rate of e-h pairs and plot them as functions of the wave vector in Fig. 5. In Fig. 5(a–d) we fix the wavevector component *k*_*y*_ = −7.5 × 10^−4^ *π*/*a* accounting for the aforementioned Dirac cone shift in k space. The energies of the e-h pairs show trigonometric-function-like shapes with respect to the wave vector *k*_*x*_ when magnetic field *B* = 1*T*, the RSOC strength *R*_0_ = 0 as read in Fig. 5(a). The wavefunction overlap of the ground-state (the lowest energy state of e-h pair) is isotropic in *k*_*x*_ (see the inset in Fig. 5(a)). Consequently, the transition rate of ground-state of e-h pair with magnetic field is isotropic as shown in Fig. 5(b). The transition rate depends on the transition matrix elements and the overlap of wavefunctions. The transition matrix elements in Eq. 7 are rather small when the RSOC terms are not included. The distribution of electrons in the ground state is greatly broadened as shown in Fig. 3(e), resulting in a relatively smaller overlap of the wavefunction. We can find that the transition rate spectrum of the ground-state e-h pairs is almost dark. As the RSOC term is considered, each energy level splits into two lines as shown in Fig. 5(c) due to the coupling of spin-up and spin-down states. Though the RSOC term further suppresses the electron distribution (see Fig. 3(e)), the coupling increases the transition matrix elements and thus increases the transition rate greatly. Therefore the RSOC term can effectively turn the transition rate spectrum of the ground-state of e-h pairs much brighter as shown in Fig. 5(d). Next, we plot the energies and the transition rates of e-h pairs as functions of wave vector *k*_*y*_ in Fig. 5(e–h). Since the periodic magnetic field destroys the time-reversal symmetry, the energies and transition rates are anisotropy as shown in Fig. 5(e) and (f), in which the transition rate spectrum of ground state is bright when momentum *k*_*y*_ is negative but dark when momentum *k*_*y*_ is positive. This feature is straightforwardly arising from the anisotropic electron distribution with different *k*_*y*_ as shown in Fig. 3(f). Accounting for that the strength of transition rate is proportional to the overlap of electron distribution and different *k*_*y*_ result in different electrons distribution(inset of Fig. 5(e)). The anisotropic transition rate spectrum from numerical calculation agrees with the theory very well. When the RSOC term is taken into consideration, we also find the spin splitting in the energy spectrum of e-h pairs as shown in Fig. 5(g). The RSOC term affects the band coupling, so the optical transition rates of ground-state become brighter in Fig. 5(h). We notice the band gap opened by the magnetic field is relatively small.

**Figure 6:**
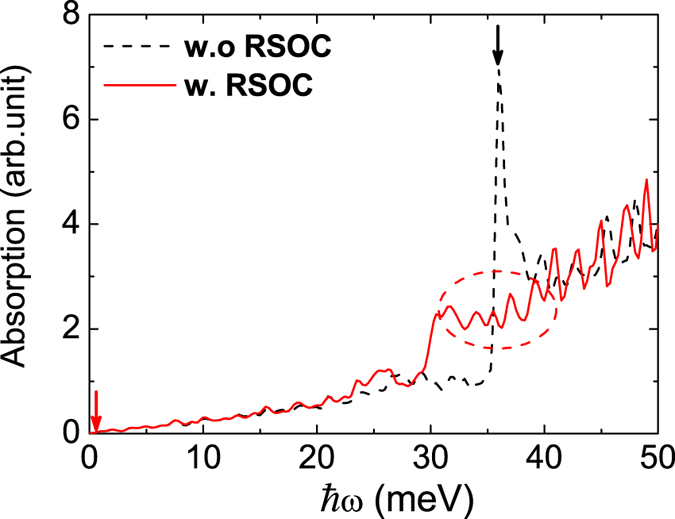
The optical absorption spectrum for the graphene magnetic supperlattice. Black dashed line without RSOC term; Red solid line with RSOC term.

Finally the optical absorption spectrum of such a graphene magnetic supperlattice is shown in Fig. 6. In this calculation, we set the fermi level at zero energy which is between the conduction and valence band. It means the occupation for the valence band is full, while that for the conduction band it is empty. We use broadening factor of 0.15 *meV* to smoothen the absorption spectrum. The optical absorption spectrum indicates useful band structure information guaranteed by the selection rule expressed as *δ*(*E*_*f*_ − *E*_*i*_ − *ħω*) in Eq. 7. The red arrow in the left of Fig. 6 denotes the vanishing optical absorption with small incident light frequency, which indicates a quite small bandgap opened by the magnetic superlattice as sketched in Fig. 1 (bottom) as well as in Fig. 2(f) and (h). Increasing the frequency of the incident light, we find a major absorption peak around *ħω*_0_ = 35 *meV* in the absence of the RSOC as denoted by the black arrow. This absorption peak agrees well with the optical transition rate diagram and is associated with the second subband. When the RSOC term is incorporated, the corresponding energy spectrum and transition rates shown in Fig. 5(c,d and g,h) can also be investigated by experimental measurement of absorption spectrum. We find that the major absorption peak splits into several sub-peaks with weaker strength but locates in a wider range as marked with the dashed circle. The appearance of sub absorption peaks manifests the band splitting the due to the RSOC term ±*R*_0_ *p*_±_ coupling spin-up and spin-down states as we discussed in the subsection Energy Spectrum. The distinct optical absorption spectra provide an effective way to control the optical properties by tuning the strength of the RSOC and magnitude of the magnetic field on graphene. The graphene magnetic supperlattice is a promising platform for potential application in anisotropic magneto-optical devices.

## Conclusions

In summary, we have investigated theoretically the energy spectrum, conductance and optical absorption spectrum of a graphene magnetic superlattice in the presence of the RSOC. We find that a finite gap is opened at the shifted Dirac point of the magnetic superlattice. The RSOC term splits the spin-degenerated energy level giving rise to a double Dirac cone. The group velocity, effective mass and charge distribution can be tuned by the magnetic fields and the RSOC effect. Importantly, anisotropic bright-to-dark transitions and peak splitting behavior in the transition rate spectra of e-h pairs are developed in the two directions respectively. As a clue for possible experimental verifications, our theoretical results demonstrate that the magnetic superlattice geometry and the RSOC effect play crucial and adjustable roles on the exotic massive spin-split states which determine the conductance gap and multiple peaks in optical absorption spectrum respectively. Our theoretical results shed new light on designing graphene-based magneto-optical devices.
